# A retail audit of mosquito control products in Busia County, western Kenya

**DOI:** 10.1186/s12936-021-03695-1

**Published:** 2021-03-23

**Authors:** Prisca A. Oria, Vincent Moshi, Julius I. Odero, Sheila Ekodir, April Monroe, Steven A. Harvey, Eric Ochomo, Danielle Piccinini Black

**Affiliations:** 1 Centre for Global Health Research (CGHR), Kenya Medical Research Institute (KEMRI), Kisumu, Kenya; 2 Johns Hopkins Center for Communication Programs, Baltimore, MD USA; 3 Johns Hopkins Bloomberg School of Public Health, Baltimore, MD USA

**Keywords:** Malaria, Spatial repellent, Mosquito control, Retail audit, Retail outlets, Insecticide‐treated mosquito nets, Aerosol sprays, Mosquito coils, Topical repellents, Kenya

## Abstract

**Background:**

Approximately 70% of Kenya’s population is at risk for malaria. The core vector control methods in Kenya are insecticide-treated mosquito nets (ITNs) and indoor residual spraying, with supplementary larval source management. In 2015, 21% of ITNs were accessed through the private retail sector. Despite the private sector role in supplying mosquito control products (MCPs), there is little evidence on the availability, sales trends, and consumer preferences for MCPs other than ITNs. This study, a component of a larger research programme focused on evaluating a spatial repellent intervention class for mosquito-borne disease control, addressed this evidence gap on the role of the private sector in supplying MCPs.

**Methods:**

A cross-sectional survey was deployed in a range of retail outlets in Busia County to characterize MCP availability, sales trends, and distribution channels. The questionnaire included 32 closed-ended and four open-ended questions with short answer responses. Descriptive analysis of frequency counts and percentages was carried out to glean insights about commercially available MCPs and the weighted average rank was used to determine consumer preferences for MCPs. Open-ended data was analysed thematically.

**Results:**

Retail outlets that stocked MCPs commonly stocked mosquito coils (73.0%), topical repellents (38.1%), aerosol insecticide sprays (23.8%) and ITNs (14.3%). Overall, retailers reported the profits from selling MCPs were adequate and they overwhelmingly planned to continue stocking the products. Of respondents who stocked MCPs, 96.8% responded that sales increased during long rains and 36.5% that sales also surged during short rains. ITNs and baby-size nets were often delivered by the wholesaler. Retailers of aerosol sprays, mosquito coils, and topical repellents either collected stock from the wholesaler or products were delivered to them. Other commercially available MCPs included insecticide incense sticks, electric mosquito strikers, insecticide soaps, electrically heated insecticide mats, and electric insecticide emanators, indicating a well-established market.

**Conclusions:**

The wide range of MCPs in local retail outlets within the study area suggests the need and demand for mosquito control tools, in addition to ITNs, that are affordable, easy to use and effective. The presence of a wide range of MCPs, is a promising sign for the introduction of a spatial repellent intervention class of products that meets consumer needs and preferences.

## Background

Despite global efforts to reduce malaria prevalence and incidence, malaria remains a major global health concern. In 2019, an estimated 229 million cases and 409,000 malaria deaths occurred worldwide with 94% of cases and deaths occurring in sub-Saharan Africa [1]. In Kenya, approximately 70% of the population is at risk for malaria and according to the 2015 Kenya Malaria Indicator Survey (KMIS), prevalence in the western lake endemic region was 27% [2]. Approximately 3 million malaria cases occurred in Kenya in 2019 [1].

Vector control is a key malaria prevention intervention and is one of the primary malaria prevention measures in Kenya. According to the Kenya National Malaria Strategy 2019–2023, the core vector control methods are insecticide-treated mosquito nets (ITNs) and indoor residual spraying (IRS), with supplementary larval source management implemented on a small scale. IRS was first implemented in Kenya from 2008 to 2012 using pyrethroids, but was not implemented from 2013 to 2016 following widespread vector resistance to pyrethroids [3]. Following the registration of an organophosphate insecticide in 2016, IRS resumed and was last deployed in 2019 in Homabay and Migori counties. Larviciding has been implemented in a few research-based small-scale trials in western Kenya [4, 5].

The Division for National Malaria Programme distributes ITNs through mass campaigns every three years in malaria endemic and epidemic-prone areas to achieve universal coverage (one net per two people) [3]. Other channels of ITN distribution include antenatal care and child welfare clinics for pregnant women and young children, social marketing at designated locations, and retail sale through commercial outlets [2]. While these distribution channels have broadened coverage, achieving and maintaining universal coverage remains a challenge due the inaccessibility of ITNs for the at-risk population in its entirety and loss of physical integrity of ITNs over time [6, 7]. In addition to the community directed mosquito control efforts organized by the government with proven efficacious interventions, there exists a considerable market for other mosquito control products (MCPs) including but not limited to mosquito coils, aerosol sprays, and topical repellents. This market demand exists even though evidence of malaria prevention efficacy of these products is lacking.

Retail markets are key malaria control product suppliers in many developing countries [8–10], and retail audits help estimate market trends. Audits collect data on sales trends, stock, supply chains, and competition. In malaria research, audits have focused primarily on medicines and ITNs [9–14], ignoring other MCPs like mosquito coils, aerosol sprays, and topical repellents. Despite free distribution efforts, supermarkets and other private shops supply 21% of Kenya’s ITNs, [2] demonstrating the retail sector’s importance for net acquisition. However, except for nets, minimal data is available on MCPs in retail outlets. Kenya’s only two previous studies examining the role of shops in mosquito control found that many households employ multiple MCPs [12, 15], often including coils and sprays in addition to ITNs.

This retail audit was a precursor to a larger research programme known as AEGIS or Advancing Evidence for the Global Implementation of Spatial Repellents. AEGIS will conduct a clinical trial to test the efficacy of a newly developed spatial repellent—Mosquito Shield™—for malaria control. Details of that trial (clinical trial registration pending) will be provided elsewhere. Mosquito Shield™ consists of a plastic sheet infused with a long-lasting formulation of transfluthrin, a World Health Organization (WHO) approved insecticide. It is intended as a supplement to, not a replacement for, ITNs. Once installed, Mosquito Shield™ releases microscopic transfluthrin particles into the air over a fixed period of time to repel or kill mosquitoes. While Mosquito Shield™ is not currently envisioned as a retail product, use of other MCPs available on the retail market could affect its acceptance among end-users. Thus, this retail audit aimed to understand the current local mosquito control landscape from a market angle through a descriptive cross-sectional survey.

## Methods

### Study site and population

This study was carried out in Teso South and Teso North sub-counties (0° 36′ 25.2″ N, 34° 16′ 33.6″ E) which cover 559 km^2^ of Busia County in western Kenya [16, 17]. The average altitude of the area is 1208 m above sea level [18]. The mean annual rainfall ranges between 800 and 1700 mm in most parts of the sub-counties, while other parts receive up to 2000 mm [16]. Temperatures are homogenous with an annual mean maximum between 26 and 30 °C and mean minimum between 14 and 22 °C [19].

The population of the area is predominantly of the Iteso ethnic group. The population at risk for malaria in the two sub-counties is the combined total population of approximately 306,150 people, according to the 2019 Kenya Population and Housing Census. Subsistence farming and small-scale trade are the dominant economic activities carried out by the inhabitants. The area is part of the lake endemic region, which has intense malaria transmission throughout the year, with peaks during the long and short rainy seasons, which generally occur in May to July and October to December [2]. ITNs have, and continue to be, distributed freely there with the aim of universal coverage, and as such, are the main interventions used by residents against malaria vectors.

### Study procedures

To develop a sampling frame, field workers conducted a census of all retail outlets in the study area using GPS-enabled tablets. Names and telephone numbers of each outlet’s attendant or owner were also noted along with whether the outlet currently stocked or had previously stocked MCPs. Outlets were categorized as either *duka* (small retail shop), pharmacy, agrovet (supply store for agricultural and veterinary products), supermarket, market stall, hawker (mobile seller) or other.

**Fig. 1:**
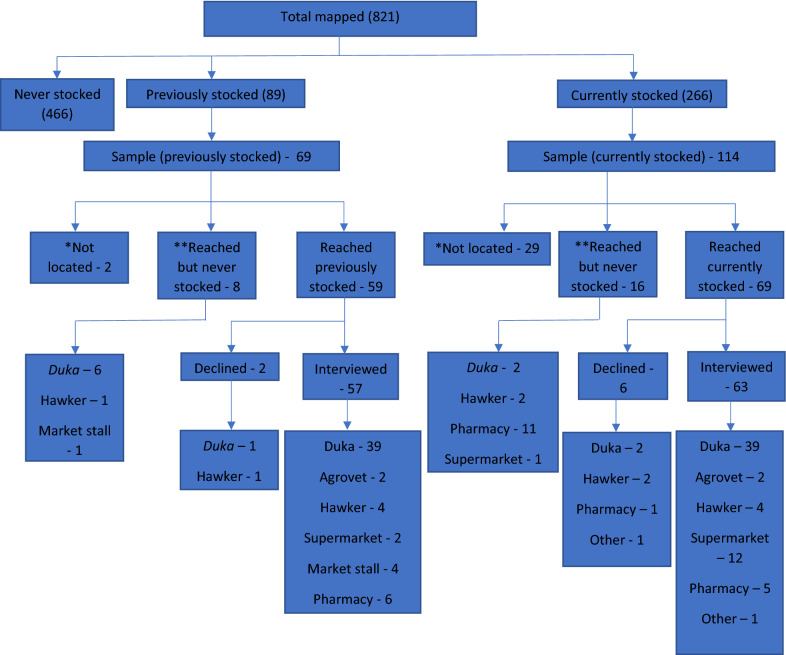
Retail outlets mapped, sampled, and audited in the study area. *Not located: the study team was unable to locate retailer for interview following inclusion in initial mapping. **Reached but never stocked: retailer was misclassified during the initial mapping as currently or previously stocked, but when contacted for interview it was determined that they had never stocked

To ensure a sample representative of all retail outlets in the study area, the study used stratified random sampling based on the census. Quotas were set for different outlet types and whether the outlet currently stocked or reported previously stocking MCPs. Outlets observed to have MCPs on the day of the census were categorized as “currently stocking.” If no MCPs were present on the day of the census, the enumerator asked the shop attendant or owner whether they had stocked MCPs at some point in the past. Those who responded “yes” were categorized as “previously stocked,” while those who responded “no” were categorized as “never stocked” (Fig. 1). Initially the study team intended to sample 50 retail outlets in each category yielding a total sample of 250. However, the census revealed that *duka* currently stocking MCPs and *duka* that had previously stocked MCPs were the only categories with more than 50 outlets. Thus, a random sample of 50 outlets was selected from each of the two *duka* categories using a random number generator. For other categories, the study team attempted to include all outlets in each category.

### Data collection

A structured tablet-based questionnaire was used to facilitate audit interviews. The questionnaire included 32 closed-ended questions and four open-ended short answer questions. Questions covered information on the role of the person interviewed, types of MCPs sold, sales trends, seasonal variations in sales, stock levels, supply chains, pricing structure, profit margins, factors affecting demand, and customer preferences as perceived by the interviewee in each outlet. The questionnaire was available in English, Kiswahili, and Ateso. Respondents selected their preferred language. The questionnaire was pre-tested in six retail outlets not included in the study before a final version was developed.

Fieldworkers fluent in the local language and with social science research training and experience collected data. The fieldworkers were trained on the content of the questionnaire, consenting procedures, research ethics and collection and transfer of data using the tablet before beginning fieldwork. Only participants 18 years and older who were either owners, managers or attendants of the retail outlet were interviewed.

During the interview process, interviewers requested to see and photograph MCPs stocked in the retail outlet. This form of observation provided triangulation for the information reported by the participant. The term ITN was used for nets treated with insecticides, UTNs for those without indications of insecticide treatment, and bed nets for a potential mix of insecticide-treated and untreated nets.

### Data analysis

Interview data was captured using CommCare 2.47.4 (Dimagi, Inc., Cambridge, MA, USA) and later downloaded into Microsoft Excel 2003 (Microsoft, Washington, USA) using CSV file format. The quantitative data was cleaned, validated, and translated into English if originally collected in Kiswahili or Ateso. The cleaned data was transferred to STATA 13 (StataCorp, College Station, Texas, USA) where descriptive analysis of frequency counts and percentages was conducted. Weighted average rank $\frac{{\sum }_{r}{n}_{r}*r}{\sum _{r}{n}_{r}}$ was used to determine customer preferences of MCPs based on participants’ knowledge of their own sales and those of other retail outlets.

Translated short answer data was transferred to Microsoft Word where two team members independently created codes by hand based on the questionnaire and data. They discussed the differences in their codebooks and then reconciled them into a single codebook. The codebook included reasons for surges and dips in sales, reasons retailers stopped selling, and factors that affected the demand for MCPs. Two other team members coded the interview data. A team member collated data relevant to each code, collated codes into themes and drafted the report.

## Results

### Retail outlets in the study area

A total of 821 retail outlets were identified and mapped in the study area census. This included 266 that reported currently stocking MCPs, 89 that reported previously stocking MCPs, and 466 that reported never having stocked MCPs (Fig. 1). *Dukas* (66.38%) constituted the largest number of retail outlets, while agrovets (2.19%) were the fewest. Other outlets included: hawkers (10.72%), market stalls (8.77%), pharmacy (5.60%), supermarkets (2.31%), and others such as wholesalers, hair salons, and barber shops (4.02%).

Of the 89 outlets reported in the census as previous MCP vendors, 69 were sampled through a combination of random sampling of *dukas* plus inclusion of all representatives of other outlet types. Field workers were unable to locate 2 of these and 8, when visited for the audit, said they had never stocked MCPs. Of the remaining 59, 2 declined to participate in the audit leaving a final sample of 57 former MCP vendors. Of the 266 identified as current MCP vendors during the census, 114 were sampled through a combination of random sampling of *dukas* plus inclusion of all representatives of other outlet types. Field workers were unable to locate 29 of these and 16, when visited for the audit, said they had never stocked. Of the remaining 69 outlets, 6 declined to participate in the audit leaving a final sample of 63 current MCP vendors. Thus, the overall sample audited included 57 former MCP vendors and 63 current vendors, for a total sample of 120.

### Characteristics of retail outlets where interviews were conducted

Most (78.9%) interview participants were retail outlet owners; others were store managers (14.3%) and store attendants (6.8%). Interviews were conducted in 120 retail outlets, 78 (65%) of which were *dukas*, as shown in Table 1.

**Table 1:** Characteristics of retail outlets identified in census (n = 821) and interviewed (n = 120)

1. Type of outlet	2. Total outlets identified in study area	3. Study area outlets that currently stocked MCPs	4. Interviewed outlets that currently stocked MCPs	5. Study area outlets that reported previously stocking MCPs	6. Interviewed outlets that reported previously stocking MCPs	7. Total interviewed (4 + 6)
	n	n (%)	n (%)	n (%)	n (%)	n (%)
*Duka*	545	202 (37.1)	39 (19.3)	70 (12.8)	39 (55.7)	78 (14.3)
Supermarket	19	13 (68.4)	12 (92.3)	3 (15.8)	2 (66.7)	14 (73.7)
Pharmacy	46	27 (58.7)	5 (18.5)	2 (4.4)^b^	6^b^	11 (23.9)
Hawker	88	15 (17.1)	4 (26.7)	7 (8.0)	4 (57.1)	8 (9.1)
Market stall	72	3 (4.2)	0 (0.0)	5 (6.9)	4 (80.0)	4 (5.6)
Agrovet	18	4 (22.2)	2 (50.0)	1 (5.6)^b^	2^b^	4 (22.2)
Other^a^	33	2 (6.1)	1 (50.0)	1 (3.0)	0 (0.0)	1 (3.0)
Total	821	266 (32.4)	63 (23.7)	89 (10.8)	57 (64.0)	120 (14.6)

^a^ Wholesaler, boutique, electronic shop, hotel, kiosk, salon, shoe store, tailor store, utensils store, welding store, barber shop, movie hall, mobile telephone handset shop

^b^Numbers interviewed are greater than numbers mapped for pharmacy and agrovet due to misclassification of outlets during initial mapping as currently or previously stocked

### MCPs stocked in retail outlets currently and previously stocking

As shown in Table 2, retail outlets that currently stocked MCPs commonly stocked mosquito coils (73.0%), topical repellents (38.1%), aerosol insecticide sprays (23.8%) and ITNs (14.3%). Outlets that previously stocked MCPs had frequently stocked mosquito coils (73.7%), ITNs (29.8%), and topical repellents (26.3%).

**Table 2:** Mosquito control products stocked in retail outlets in interview sample (n = 120)

Retail outlet type	Stocking status	Insecticide-treated nets	Baby nets	Aerosol insecticide sprays	Mosquito coils	Topical repellents	Other^b^
		n (%)	n (%)	n (%)	n (%)	n (%)	n (%)
*Duka*	Currently n = 39	4 (10.3)	0	4 (10.3)	33 (84.6)	12 (30.8)	15 (38.5)
	Previously n = 39	9 (23.1)	0	2 (5.1)	34 (87.2)	8 (20.5)	2 (5.1)
Pharmacy	Currently n = 5	0	0	0	0	3 (0.6)	4 (0.8)
	Previously n = 6	3 (50.0)	0	2 (33.3)	2 (33.3)	3 (50.0)	1 (16.7)
Supermarket	Currently n = 12	5 (41.7)	3 (25.0)	9 (75.0)	11 (91.7)	6 (50.0)	11 (91.7)
	Previously n = 2	0	0	0	2 (100.0)	1 (50.0)	1 (50.0)
Hawker	Currently n = 4	0	0	1 (25.0)	2 (50.0)	2 (50.0)	0
	Previously n = 4	2 (50.0)	0	0	3 (75.0)	1 (25.0)	0
Market stall	Currently n = 0	0	0	0	0	0	0
	Previously n = 7	3 (42.9)	0	1 (14.3)	1 (14.3)	2 (28.6)	0
Agrovet	Currently n = 2	0	0	1 (50.0)	0	0	1 (50.0)
	Previously n = 2	0	0	0	0	0	2 (100.0)
Other^a^	Currently n = 1	0	0	0	0	1 (100.0)	0
	Previously n = 0	0	0	0	0	0	0
Total	Currently n = 63	9 (14.3)	3 (4.8)	15 (23.8)	46 (73.0)	24 (38.1)	31 (49.2)
	Previously n = 57	17 (29.8)	0	5 (8.8)	42 (73.7)	15 (26.3)	6 (10.5)

^a^Wholesaler

^b^Untreated bed nets, residual insecticide sprays, electric mosquito strikers, malaria prophylaxis, insecticide incense sticks, insecticide soap, electric mosquito mats, electric mosquito repellent, insect killer, mosquito repellents and insecticide candles

### Product supply chain and frequency of restocking in retail outlets that currently stocked MCPs

ITNs and baby-size nets were mainly restocked monthly or depending on sales and were most often delivered to the retail outlet by the wholesaler with upfront cash payment, as shown in Table 3. Retailers of aerosol sprays, mosquito coils, and topical repellents often paid cash upfront to the wholesaler for stock, which they either picked up from the wholesaler or products were delivered to them.

**Table 3:** Frequency, mode, source and payment method for retail outlets that currently stocked mosquito control products (n = 63)

Stocking and supply chain	Mosquito control product	ITN n = 9	Baby-sized bed net n = 3	Aerosol insecticide sprays n = 16	Mosquito coils n = 45	Topical repellents n = 24	Other^c^ n = 19
		n (%)	n (%)	n (%)	n (%)	n (%)	n (%)
Frequency of stocking	Weekly	0	0	2 (12.5)	7 (15.6)	3 (12.5)	3 (15.8)
	2 weeks	0	0	2 (12.5)	7 (15.6)	0	2 (10.5)
	Monthly	5 (55.6)	2 (66.7)	6 (37.5)	12 (26.7)	11 (45.8)	3 (15.8)
	> Monthly	2 (22.2)	0	3 (18.8)	5 (11.1)	3 (12.5)	1 (5.3)
	Depends on sales	2 (22.2)	1 (33.3)	3 (18.8)	13 (28.9)	4 (16.7)	5 (26.3)
	Other^a^	0	0	0	1 (2.2)	3 (12.5)	5 (26.3)
How stock was obtained	Delivered	6 (66.7)	3 (100.0)	14 (87.5)	22 (48.9)	11 (45.8)	17 (89.5)
	Pickup	3 (33.3)	0	2 (12.5)	23 (51.1)	13 (54.2)	2 (10.5)
Where stock was obtained	Wholesaler	9 (100.0)	2 (66.7)	15 (93.8)	43 (95.6)	24 (100.0)	18 (94.7)
	Other^b^	0	1 (33.3)	1 (6.2)	2 (4.4)	0	1 (5.3)
How Retailers Paid for stock	On consignment	2 (22.2)	1 (33.3)	5 (31.2)	5 (11.1)	2 (8.3)	3 (15.8)
	Full Value	7 (77.8)	2 (66.7)	11 (68.8)	40 (88.9)	22 (91.7)	16 (84.2)

^a^Customer request, depends on season, depends on supplier, don’t know and first stock

^b^Pharmacy, retailer, distributor, don’t know

^c^Untreated bed nets, electric mosquito mat, electric mosquito repellent, malaria prophylaxis, insect killer, mosquito repellents, mosquito candles, soap, insecticide incense sticks, electric mosquito striker, residual insecticide spray

### Profit adequacy and plans to continue stocking MCPs

Overall, retailers that currently stocked MCPs reported the profits were adequate and they overwhelmingly planned to continue stocking the products, as shown in Table 4. Of retailers who stocked ITNs, 55.6% (5 out of 9) reported that the profit was adequate. All respondents stocking ITNs, UTNs, baby-sized nets, aerosol insecticide sprays, and residual insecticide spray planned to continue stocking the products. Of those stocking mosquito coils and topical repellents, 97.8% (44 out of 45) and 91.7% (22 out of 24), respectively, planned to continue stocking the products. A couple (2 out of 24) retailers of topical repellents planned to stop stocking the products.

**Table 4:** Profit margins, adequacy and plans to keep stocking products (n = 63)

Mosquito control product	Profit margin (Kenya Shillings)	Perception of profit			Keep selling		
		Adequate	Inadequate	Other^a^	Yes	No	Don’t know
	(min, max)	n (%)	n (%)	n (%)	n (%)	n (%)	n (%)
ITN (n = 9)	(50, 200)	5 (55.6)	4 (44.4)	0	9 (100.0)	0	0
UTN (n = 1)	(50, 50)	1 (100.0)	0	0	1 (100.0)	0	0
Baby-sized bed net (n = 3)	(50, 50)	1 (33.3)	1 (33.3)	1 (33.3)	3 (100.0)	0	0
Aerosol insecticide sprays (n = 16)	(20, 265)	11 (68.8)	5 (31.2)	0	16 (100.0)	0	0
Mosquito coils (n = 45)	(0.5, 165.5)	30 (66.7)	14 (31.1)	1 (2.2)	44 (97.8)	0	1 (2.2)
Topical repellents (n = 24)	(5, 48)	15 (62.5)	8 (33.3)	1 (4.2)	22 (91.7)	2 (8.3)	0
Other^b^ (n = 18)	(3, 150)	13 (72.2)	5 (27.8)	0	15 (83.3)	2 (11.1)	1 (5.6)

^a^Neither adequate nor inadequate

^b^Electric mosquito mat, electric mosquito repellent, insect killer, malaria prophylaxis, mosquito repellents, mosquito candles, soap, insecticide incense sticks, electric mosquito striker, and residual insecticide spray

### Ranking of stocked MCPs when more than one product was stocked

Based on retailers’ own MCP sales and their knowledge of other retail outlets’ sales, study participants ranked mosquito coils, topical repellents, aerosol insecticide sprays and ITNs as the products most frequently purchased by customers in outlets that stocked more than one MCP, as shown in Table 5.

**Table 5:** Ranking of most frequently sold mosquito control products when more than one product was stocked

Mosquito control product	1st (most commonly sold)	2nd	3rd	4th	5th	6th (least commonly sold)	By weighted rank
	n	n	n	n	n	n	n
ITN (n = 8)	3	1	4	–	–	–	3
Baby-sized bed net (n = 3)	–	–	–	1	1	1	6
Aerosol insecticide sprays (n = 13)	6	4	2	1	–	–	2
Mosquito Coils (n = 23)	15	8	–	–	–	–	1
Topical Repellents (n = 19)	2	10	3	3	1	–	4
Other^a^ (n = 14)	1	4	4	2	2	1	5

^a^UTN, electric mosquito mat, electric mosquito repellent, insect killer, mosquito repellents, mosquito candles, soap, insecticide incense sticks, electric mosquito striker, and residual insecticide spray

When the ranking by retailers was weighted, mosquito coils, aerosol insecticide sprays, ITNs, and topical repellents were perceived by retailers as the products most frequently purchased by consumers in that order.

### Additional MCPs used by residents and their sources based on retailer reports

In addition to the MCPs they sold, retailers also reported that consumers used products obtained elsewhere. These products included ITNs (49), aerosol sprays (16), topical repellents (14) and mosquito coils (11), among other products, as shown in Table 6.

**Table 6:** Additional mosquito control products used by residents and their sources based on retailer reports (n = 63)

Product	Frequency	Where product was obtained					
		Free distributions	Hospital/health centres	Retail shop	Homemade	Supermarket	Other^a^
	n	n (%)	n (%)	n (%)	n (%)	n (%)	n (%)
Aerosol sprays	16	1 (6.3)	–	15 (93.8)	–	4 (25.0)	1(6.3)
Bed nets	49	29 (59.2)	32 (65.3)	29 (59.2)	–	2 (4.1)	1 (2.0)
Electric emanator	3	–	–	2 (66.7)	–	2 (66.67)	0
Electric mosquito striker	8	1 (12.5)	–	7 (87.5.)	–		1 (12.5)
Leaves/smoke	8	–	–		7 (85.7)		1 (12.5)
Mosquito coils	11	–	–	11 (100.0)	–		
Topical repellents	14	–	–	13 (92.9)	–	2 (14.3)	2 (14.3)
Other^b^	6	–	–	5 (83.3)	–	1 (16.7)	–

A type of product could reportedly be obtained from different sources, i.e., because bed nets can be obtained from mass distribution and retail outlets the percentages add up to more than 100

^a^Pharmacy, hawkers, wholesaler, and home-made

^b^Indoor residual spray, insecticide incense sticks, soap, celling fan, blust bulb

The highest proportion (65.3%) of ITNs were reportedly obtained from antenatal and immunization clinics, 59.2% from free mass distribution and another 59.2% purchased from retail outlets. Mosquito coils were reportedly exclusively purchased from retail outlets. Topical repellents were mainly (92.9%) obtained from retail outlets as were aerosol sprays (93.8%). A type of product could reportedly be obtained from different sources, i.e., because bed nets can be obtained from mass distribution and retail outlets the percentages add up to more than 100.

### Seasonal variation in sales of MCPs

Of respondents who currently stocked MCPs, 96.8% (61 of 63) responded that sales surged during long rains and 36.5% (23 of 63) informed sales also surged during short rains. Fifty-seven (98.3%) respondents reported a dip in sales of MCPs during the dry season.

#### Reasons for surges and dips in sales of MCPs

The main reason mentioned for increased sales of MCPs was an increase in mosquito numbers mainly a result of an increase in breeding grounds from stagnant rainwater. Other reasons for increased mosquito numbers included: longer grass, presence of swamps and increase in mosquito breeding. Student purchases for overnight field trips, increased awareness of mosquito control and increased household population, also led to a surge in sales.

The main reasons mentioned for decreased sales of MCPs was a reduction in number of mosquitoes or absence of mosquitoes. Both a reduction and absence of mosquitoes were mainly attributed to increased heat or sunny weather, fewer/lack of breeding grounds as stagnant rainwater dried up and reduction in bushes as crops were harvested. Other reasons for a reduction in sales of MCPs were free distribution of ITNs, few malaria cases, and absence of mosquitoes due to dry weather conditions. Decrease in malaria was associated with fewer or no mosquitoes in houses.

### Factors that affected the demand for MCPs based on retailer reports

#### Factors reported to affect the demand for MCPs

**Fig. 2:**
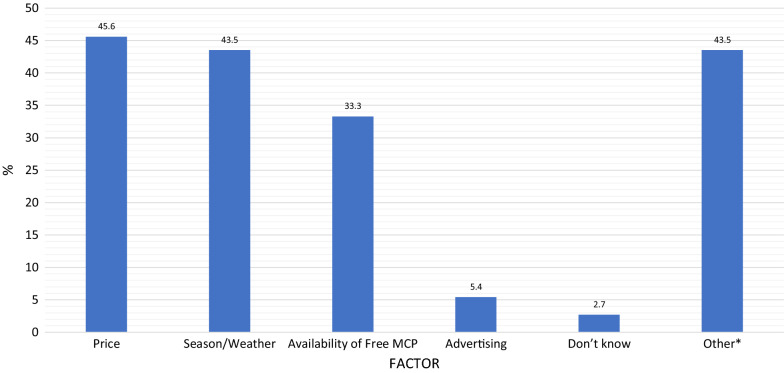
Factors that affected demand for mosquito control products. *Other responses are detailed in the sections below on factors reported to negatively and positively affect the demand for MCPs

When asked what factors influenced demand for MCPs, retailers mentioned price most frequently (45.6%), followed closely by season/weather (43.5%). These results are presented in Fig. 2. A considerable number of retailers had different responses (43.5%), apart from what were provided as likely choices, which were collected qualitatively and are presented in the sections below.

#### Factors reported to negatively affect the demand for MCPs

The main factors that retailers reported to negatively affect demand for MCPs were adverse effects from product use, perceived ineffectiveness, inconvenience of use, short period of product use and inaccessibility. Adverse effects included bad smelling fumes, eye irritation, allergic reactions and coughs from burning mosquito coils; skin irritation from new ITNs; skin reactions and rashes from topical repellents; and the perception that ITNs introduced bed bugs in homes.

Retailers reported that generic products were perceived by consumers as inferior in quality to brand name products, which influenced demand for particular brands of products. Inconveniences for bed net use such as time needed to hang them and inability to carry them to funerals or overnight school field trips were perceived to have a negative effect on demand. The burden of topical repellents being easily wiped off by blankets and mosquito coils breaking easily and running out quickly were also perceived to negatively affect demand. Retailers also lacked products that were difficult to source or that suppliers had not delivered resulting to lost consumer interest. Other factors reported to negatively affect the demand for MCPs were lack of money to buy enough bed nets for the whole family and the need for electricity to power some MCPs.

#### Factors reported to positively affect the demand for MCPs

For MCPs generally, perceived effectiveness and trust in the product, increase in mosquitoes and malaria, and fear of mosquito bites were perceived as positively affecting demand. Customers specifically liked bed nets because they have a longer life of use.

### Retailer reasons for no longer stocking MCPs

Low demand, customer complaints about adverse effects, and difficulties obtaining stock were the main reasons retailers did not stock MCPs when this study was conducted. Low demand was attributed to free distribution of ITNs in health facilities and people having ITNs. Retailers reported that customers often complained that burning coils emitted a bad smell and caused coughs and flu. Some customers did not like the smell of topical repellents. Sometimes shops had not restocked because either obtaining stock was difficult or their supplier had not delivered stock. Other reasons retailers had no stock included lack of money to restock and yet to restock sold-out products.

Additional reasons retailers did not stock MCPs were customer complaints about perceived ineffectiveness, lack of capital, had stopped selling MCPs and had closed business.

## Discussion

This study assessed the availability, supply chains, and sales trends of MCPs within the area selected for the AEGIS efficacy trial. While Mosquito Shield™, the spatial repellent to be assessed in the forthcoming clinical trial, is not currently intended for retail sale, the existing retail market for MCPs is likely to have an effect on consumer acceptance of Mosquito Shield™ and potential similar products. Knowing what MCPs retail outlets are currently selling is a first step towards understanding consumer preferences and how these preferences might affect uptake of a new product.

The audit found a wide range of MCPs on the market, mainly comprising mosquito coils, topical repellents, aerosol sprays, ITNs, and residual insecticide sprays. This widespread availability of MCPs is evidence that there is a market for such products in Busia County. While evidence shows that mosquito coils [20–23], topical repellents [24–26], and aerosol insecticide sprays [27] help reduce mosquito bites, there is no data to suggest these products reduce the incidence of clinical malaria [23, 28, 29]. Nevertheless, consumers are buying them.

In addition to these better-known MCPs, there were also insecticide incense sticks, electric mosquito strikers, insecticide soaps, electrically heated insecticide mats and electric insecticide emanators available, suggesting that the MCP market is well-enough established to support new, niche, products. While these data are cross-sectional and cannot be used to estimate market trends over time, the fact that new products are becoming available and retailers are stocking them provides some indication of market growth. The type of retailers surveyed operate on very small margins and typically cannot afford to stock products that do not sell. Therefore, the presence of these products in multiple retail outlets provides fairly strong evidence that consumers are purchasing them.

Mosquito coils, topical repellents, and aerosol insecticide sprays by design are easy to use and can provide protection against nuisance biting outside of sleeping hours. They fill a need that ITNs, which have high ownership in the study area [18] do not, thus suggesting that other MCPs still play an important role in mosquito control in this malaria endemic zone.

While ITNs provide protection indoors during sleeping hours, residents likely use additional personal protective measures for early evening indoor and outdoor mosquito biting. These findings mirror those of studies carried out elsewhere in Kenya that report household use of multiple MCPs, with sleeping under an ITN as the main method [12, 15]. In a study that assessed the role of shops in the treatment and control of childhood malaria along the coast of Kenya, 46.4% of the consumers reported using commercial pyrethrum mosquito coils, reinforcing the product’s popularity [12]. This may be related to the affordability, perceived effectiveness, and convenience of using mosquito coils to protect against early evening biting when ITNs cannot be deployed or are unavailable. According to retailers in this study, price had the most impact on demand for MCPs, closely followed by weather and free access. Rainy seasons were associated with increases in mosquito densities and may be a driving factor in seeking additional MCPs for use outside of sleeping hours. The study area is part of Kenya’s malaria-endemic lake region, but retailers perceived increases in mosquito abundance, malaria incidence and MCPs sales during rainy seasons. This is consistent with epidemiological evidence demonstrating that, while some malaria transmission occurs year-round, it peaks during the rainy seasons.

ITNs were hardly available in retail outlets despite retailers’ belief that other retailers stocked them. This is likely the result of the easy ITN access: Kenya’s National Malaria Control Programme distributes ITNs through mass campaigns every three years and routinely through antenatal care and child welfare clinics for pregnant women and young children [2]. Pharmacies, market stalls and hawkers reported previously selling ITNs but had stopped because of the low demand induced by free access. When asked which MCPs consumers used in addition to those sold in their own stores, 78% of retailers mentioned bed nets. Mosquito coils were widely available in *dukas* and supermarkets. While aerosol insecticide sprays and topical repellents were mainly found in *dukas* and supermarkets, hawkers and market stalls also stocked topical repellents.

Retailers obtain most of their MCP stock from wholesalers. Retailers of aerosol sprays, mosquito coils and topical repellents pay cash up front for stock, and products are either delivered to them or the retailer collects stock from the wholesaler. Difficulty obtaining supplies was one reason retailers often mentioned for having MCPs temporarily out of stock. Overall, however, retailers were satisfied with their profit margins from selling MCPs and planned to continue stocking them.

Regardless of whether a new spatial repellent intervention labelled for public health becomes available on the retail market, this audit makes clear that Busia County residents currently rely on retail outlets for MCPs that are complementary to ITNs now. It is essential to consider this fact when preparing to introduce such a spatial repellent into the malaria control portfolio, since currently available products could pose competition and inhibit uptake. It will be important to identify the consumer demand and preferences met by current MCPs (e.g., ease of use and use outside of sleeping hours), and position spatial repellents as a product that meets those needs more effectively and with fewer adverse effects. Another consideration for successful introduction of a novel spatial repellent is the potential power of branding. While this retail audit did not directly ask retailers about brands, a few brands for aerosol insecticide sprays, mosquito coils, and topical repellents featured repeatedly in our audit data indicating that they were top-of-mind.

## Limitations

This study had a number of limitations. Probably the most important limitation of this study was the limited sample size, a result of the limited number of retail outlets in the study area. In this study, most of the retailers identified were *dukas* and there were insufficient numbers of other retailer categories to include 50 units of each. This issue was compounded by the fact that some itinerant retailers identified in the census could not be located again for an audit interview. Some identified in the census as current or previous MCP vendors reported never having sold MCPs when invited to participate in the audit. Retailers also showed some reluctance to reveal profit margins on MCPs fearing that such information might be shared with revenue and tax authorities or competitors. Additionally, because there are no current plans to distribute the new spatial repellent through retail channels, this study did not ask about retailers’ potential willingness to stock it. Retailer willingness to stock the new spatial repellent, trends in pricing and the effect of pricing on product demand, and retailer perceptions of what would make the product more attractive to retail consumers, could be important questions for future research.

Since the objective of this study was to describe the retail landscape for MCPs in the study area, only retail vendors were interviewed. Thus, information about consumer preferences and purchasing patterns is based only on retailer reports. This means, for instance, that data is not available on whether certain classes of consumers prefer one category of retailer over another or what consumers do at any given time if their preferred retailer does not have their preferred product in stock. Likewise, the study does not provide direct information about wholesale markets or wholesale trends. Interviews with consumers and wholesalers to explore these questions would be appropriate for future research.

## Conclusions

The presence and wide range of MCPs in local retail outlets suggest that there is a market for MCPs in Busia County. This indicates that there is ongoing demand for MCPs, in addition to freely distributed ITNs, that are affordable, easy to use, and effective in situations where ITNs cannot provide protection (i.e. outside of sleeping hours both indoors and outdoors). Mosquito coils, aerosol insecticide sprays and topical repellents were the most widely sold products, with mosquito coils being the dominant product likely because they met that criteria. Additionally, the new MCPs on the market; insecticide incense sticks, electric mosquito strikers, insecticide soaps, electrically heated insecticide mats, and electric insecticide emanators, are an indication that the market for MCPs is well-established.

This well-established market is a promising sign for the introduction of a spatial repellent product class that has proven efficacy for public health and meets consumer demand and preferences, such as ease of use and continuous protection indoors, outside of sleeping hours, without notable adverse effects. Mosquito coils were popular despite the need to frequently replenish and light them, irritating smell, and fumes that caused coughs. An odourless spatial repellent at an accessible price point, that requires infrequent replacement, could prove to be a popular product and potential supplement for coils.

## Data Availability

The data sets generated and analysed during the current study are available from the corresponding author on reasonable request.

## Abbreviations

AEGIS: Advancing Evidence for the Global Implementation of Spatial Repellents
GPS: Global Positioning System
IRS: Indoor residual spraying
ITN: Insecticide-treated mosquito net
KMIS: Kenya Malaria Indicator Survey
MCP: Mosquito control product
SR: Spatial repellent
UTN: Untreated bed net
